# Magnetic-Polaron-Induced Enhancement of Surface Raman Scattering

**DOI:** 10.1038/srep19025

**Published:** 2016-01-12

**Authors:** Qi Shao, Fan Liao, Antonio Ruotolo

**Affiliations:** 1 Department of Physics and Materials Science, City University of Hong Kong, Kowloon, Hong Kong SAR, China; 2 Shenzhen Research Institute, City University of Hong Kong, High-Tech Zone, Nanshan District, Shenzhen 518057, China; 3 Jiangsu Key Laboratory for Carbon-Based Functional Materials and Devices, Institute of Functional Nano and Soft Materials Laboratory (FUNSOM), Soochow University, Suzhou, Jiangsu 215123, China

## Abstract

The studies of the effects of magnetic field on surface enhanced Raman scattering (SERS) have been so far limited to the case of ferromagnetic/noble-metal, core/shell nano-particles, where the influence was always found to be negative. In this work, we investigate the influence of magnetic field on a diluted magnetic semiconductor/metal SERS system. Guided by three dimensional finite-difference time-domain simulations, a high efficient SERS substrate was obtained by diluting Mn into Au-capped ZnO, which results in an increase of the dielectric constant and, therefore, an enhancement of Raman signals. More remarkably, an increase of intensities as well as a reduction of the relative standard deviation (RSD) of Raman signals have been observed as a function of the external magnetic strength. We ascribe these positive influences to magnetic-field induced nucleation of bound magnetic polarons in the Mn doped ZnO. The combination of diluted magnetic semiconductors and SERS may open a new avenue for future magneto-optical applications.

Surface plasmon resonance (SPR) of metal nanostructures is the source of various surface-enhancement applications, including surface-enhanced Raman scattering (SERS) and metal-enhanced-fluorescence (MEF)^1^,^2^. SERS is of particular practical interest because it allows detection of the trace level of chemicals or bio-analytes adsorbed on the nanostructured metal surfaces^3^,^4^,^5^,^6^,^7^. Due to a small fraction of inelastic scattering of the incident laser, the native weak signal of spontaneous Raman scattering did not lead to appreciable interests, until the landmark observation of the SERS phenomenon made a fascinating possibility for practical applications by an enhancement factor (EF) of 10^6^ or even larger^8^.

The SPR depends on two major factors: one is the size, inter spacing and charge densities of the nanoparticles; the other is the dielectric constant of the surrounding medium^9^,^10^. In general, for a given NP size, the interaction between the substrate and the NPs increases with decreasing gap-distance between NPs^11^. Instead, with a fixed gap, the interaction shows a non-monotonic dependence on the NP size. For instance, in a NPs/Si system with a gap of 2 nm, the SERS enhancement factor increases by up to 20 times as the particle size increases from 10 nm to 50 nm, then it starts decreasing for larger particle size, therefore indicating the existence of an optimum size of the NPs^12^.

The SPR absorption wavelength *λ* can be expressed as: *λ*^2^ = *λ*_p_^2^(*ε*_∞_ + 2*ε*_m_), where *λ*_p_ is absorption wavelength of the bulk material, *ε*_∞_ is the high-frequency dielectric constant, *ε*_m_ is the dielectric constant of the surrounding environment^13^. Thus high dielectric constants result in an enhancement of the plasma-field, which is usually accompanied by a red-shift of the SPR peak^14^,^15^. Well-defined SPR signals can be obtained by tuning the dielectric constants of a semiconductor substrate by introducing different metal dopant concentrations^16^,^17^.

Diluting magnetic dopants into wide band-gap semiconductors leads to ferromagnetic order due to interaction between localized magnetic moments mediated by free charge carriers^18^. In these so-called diluted magnetic semiconductors (DMSs), ferromagnetic domains tend to form around native donor-defects, such as oxygen vacancies, and are named magnetic polarons. The application of an external magnetic field favors the nucleation of additional magnetic polarons, hence changing the overall magnetic moment, when a DMS is used as a substrate. Unlike the previously studied core-shell systems, in which a magnetic field has been reported to have a negative influence on the SERS due to reduced electrons transfer to metal surface, as well as broadening of the energy gap^19^,^20^, a magnetic order in a DMS is expected to increase thermal stability^21^. This motivated us to investigate the effect of external magnetic fields on a diluted magnetic semiconductor/metal SERS systems.

In the current work, we theoretically and experimentally examine the SERS effect in nano-structured ZnO/Au substrates, when Mn is doped into ZnO. Finite-difference-time-domain (FDTD) calculations showed that changing the dielectric environment of ZnO by doping with Mn results in a red-shift and an enhancement of intensity (|E/E_0_|^2^). A maximum intensity (|E/E_0_|^2^) five times larger than that of pure ZnO/Au was theoretically predicated and experimentally verified for a doping of 8% Mn into ZnO. Experimentally, we compared different SERS activities using various Mn-doped nonpolar ZnO SERS substrates in an attempt to match simulation results. More remarkably, we show that, unlike the case of core-shell structure, the application of a magnetic field has a beneficial influence on the SERS signal by increasing intensities and reducing the relative standard deviation (RSD). We ascribe these effects to thermal stabilization due to magnetic field induced nucleation of bound magnetic polarons in the Mn doped ZnO layer.

## Results

**Figure 1:**
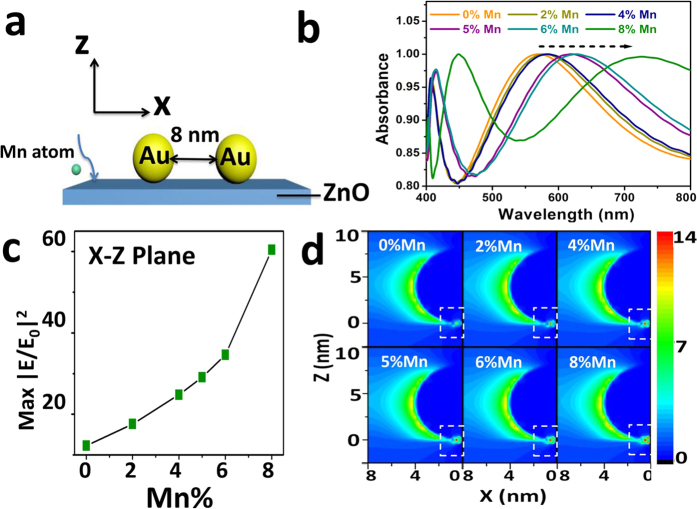
(**a**) Schematic of 3D FDTD calculation of intensity (|E/E_0_|^2^) distributions at wavelength of 633 nm with 8 nm Au nanoparticles and 8 nm interparticle separation on ZnO film; (**b**) A red shift of SPR as increasing concentrations of Mn dopants; (**c**) Maximum intensities (|E/E_0_|^2^) as a function of different Mn dopant concentrations and (**d**) Images of different intensity (|E/E_0_|^2^) distributions between Au nano-particles on different Mn (0%, 2%, 4%, 5%, 6%, and 8%)-doped ZnO substrates observed on *x-z* direction.

3D finite-difference time-domain simulations were first carried out to guide the experiments by tuning six different concentrations of Mn (0%, 2%, 4%, 5%, 6%, and 8%) diluted in the ZnO substrate. We limited our study to a maximum concentration of 8% Mn, because this is the maximum solubility of Mn into ZnO we can achieve with our thin film technology^22^. A frequency-domain field profile monitor was used to record the electromagnetic field over the simulation region. Figure 1a shows the FDTD simulation conditions. Au nanoparticles (NPs) with diameter 8 nm are assumed to be deposited on non-doped or Mn-doped ZnO with an interparticle distance of 8 nm in the *x-y* plane. Beneath the array, the pure ZnO or Mn-doped ZnO thin films have thickness of 250 nm. Periodic boundary conditions were applied to the *x*- and *y*- directions to describe an infinite array; perfectly matched layers were set to the z- boundaries as a boundary condition. A p-polarized (*λ* = 633 nm) plane wave was placed above the Au array and polarized to the x-axis. The mesh size used in the calculations was 0.25 nm × 0.25 nm × 0.25 nm. The simulation time was set at 1000 fs, ensuring the fields to decay completely before termination of the simulation.

The permittivity data of undoped and Mn-doped ZnO were calculated as *ε* = (n − *i*k)^2^ = *ε*_1_ − *iε*_2_ = (n^2^ − k^2^)–2*i*nk and are listed in Table S1, where *ε*_1_ is the real part of the permittivity and *ε*_2_ is the imaginary part. For the calculation, the refractive indices (n) of 0% and 5% Mn doped ZnO thin films were obtained from ref. 23. Those of 2%, 4%, 6% and 8% Mn-doped ZnO were calculated using Bruggeman equation^24^. The extinction coefficient (*k*) was calculated from the equation: k = *αλ*/4*π*, where the absorption spectra (*α*) were obtained from ref. 25. The experimental value of Palik was used for the dielectric constants of Au^26^.

Simulations show penetration of the electric field in two regions: between the Au NP and ZnO film (with or without Mn dopant) and in the interparticle. Considering that the near-field enhancement prefers to localize in the gap between NP and film using a 633 nm laser irradiation, *x-z* plane monitor was placed at the center of the sphere^27^. An obvious red shift with increasing Mn concentration can be observed in Fig. 1b. From Table S1, the dielectric constants of ZnO increase with increasing Mn dopants, providing a changing surrounding medium, which tunes SPR peaks to longer wavelength.

Maximum intensities (|E/E_0_|^2^) for different Mn-doped ZnO substrates are shown in Fig. 1c,d. The intensity increases more than linearly with Mn concentration. The maximum intensity (|E/E_0_|^2^) for a concentration of 8% Mn-doped ZnO/Au was 60.4, which is nearly five times larger than that of pure ZnO/Au. Since SERS signals have quartic dependence on the electric field intensity (|E/E_0_|), a much stronger SERS signal is predicted for Mn-doped ZnO/Au as compared to ZnO/Au substrate^11^. We will experimentally verify this prediction in the following.

Note that the imaginary part of the dielectric constant in Table S1 is not monotone with the concentration of Mn. It reaches a maximum for 5% Mn and then starts decreasing. This is in agreement with the accepted notion that the dominant effect leading to a monotonically increase of (|E/E_0_|^2^) is the localized interaction between the Au NPs and the ZnO substrate, which is mainly determined by the real part of the permittivity^28^.

**Figure 2:**
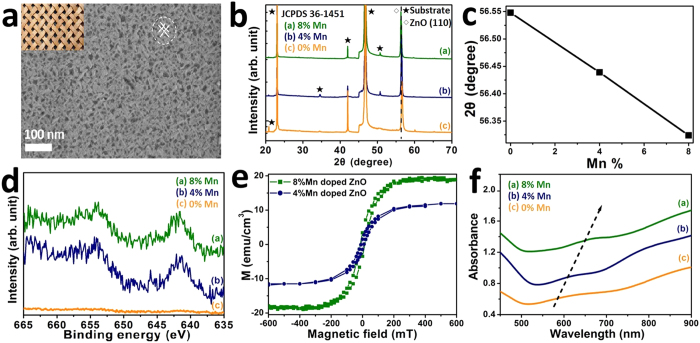
(**a**) SEM image of nonpolar ZnO. Inset: morphology of real “wood woven”; (**b**) XRD patterns of nonpolar (0%, 4%, and 8%) Mn-doped ZnO films; (**c**) (110) peak location comparisons between nonpolar (0%, 4%, and 8%) Mn-doped ZnO films; (**d**) XPS spectrums of Mn 2*p* peaks of nonpolar (0%, 4%, and 8%) Mn-doped ZnO films; (**e**) Room temperature MH-loops of nonpolar (4%, 8%) doped ZnO films; (**f**) UV-Vis spectrums of nonpolar (0%, 4%, and 8%) Mn-doped ZnO/Au films.

In order to verify the prediction of the simulations, we used our well-established technology to grow Mn-doped ZnO films with different concentration of Mn, up to 8%^22^. In brief, we exploited the lattice mismatch between a (100) Strontium Titanate (STO) wafer and ZnO to spontaneously obtain a “wood-woven” nanostructured film consisting of ordered nanorods grown along two directions, that is, one perpendicular to the other. The SEM micrograph of a typical film is shown in Fig. 2a. The morphology of the film was found to be independent on the Mn concentration. The X-ray diffraction (XRD) patterns in Fig. 2b show that the films grow along the nonpolar directions (110) and its perpendicular to the *c*-axis. A small shift towards smaller angle is observed when increasing the Mn concentration (see Fig. 2c), which is due to the slightly larger radius of the Mn^2+^ (0.066 nm) as compared to that of Zn^2+^ (0.060 nm)^22^. In Fig. 2d, X-ray photoemission spectroscopy (XPS) confirms that Mn replaces Zn with the same valence 2+ for Mn doping up to 8%. The magnetization versus magnetic field (M-H) curves of (4%, 8% Mn) doped ZnO films were measured at room temperature and are shown in Fig. 2e. All films show room temperature ferromagnetism, with a magnetic moment that increases while increasing Mn concentration. It is important to notice that the M-H loops show very little remanence and coercivity. As will be further discussed later, this is common to DMSs where the magnetic order is mostly induced by the application of an externally applied magnetic field^18^. Au NPs were deposited on the Mn-ZnO films by resorting to sputtering. While Au sputtered on plain Mn-ZnO films form a smooth and compact film, when the material is sputtered on nanostructured Mn-ZnO the highly energetic Au atoms reaching the substrate tend to fall into the valleys and to cluster in nanoparticles (see Figures S4 and S5 in Additional Information), rather than forming a continuous film. The NP size depends on the deposition time, which we calibrated to obtain NPs of 8 nm, in order to comply with the previously shown simulations. UV-vis spectroscopy was used to characterize the localized surface plasmon resonance (LSPR) properties of (0%, 4%, and 8%) Mn-doped ZnO/Au substrates. The absorbance spectra are shown in Fig. 2f. The LSPR peaks are red shifted from 580 nm to 640 nm as the Mn concentrations increase, which matches well with the simulation results in Fig. 1b.

**Figure 3:**
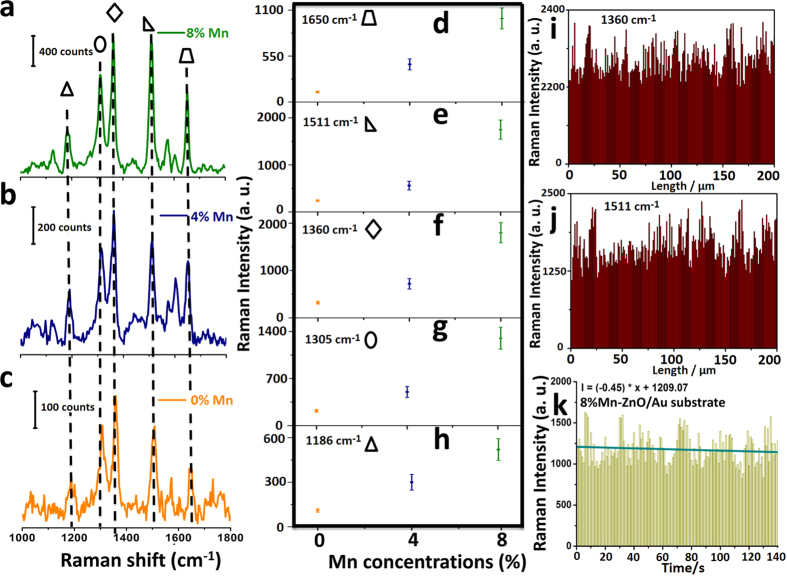
(**a**) 8% Mn doping, (**b**) 4% Mn doping, (**c**) 0% Mn doping. (**d–h**) Variations of SERS intensities of nonpolar Mn (0%, 4%, 8%) doped ZnO/Au substrates at picked peaks: (**d**) 1650 cm^−1^, (**e**) 1511 cm^−1^, (**f**) 1360 cm^−1^, (**g**) 1305 cm^−1^, (**h**) 1186 cm^−1^. (**i–j**) The intensities of the main Raman vibrations of 1 × 10^−8 ^M R6G solution for a nonpolar 8% Mn doped ZnO/Au substrate: (**i**) 1360 cm^−1^, (**j**) 1511 cm^−1^. (**k**) Intensities of 1360 cm^−1^ to measure thermal ability of a nonpolar 8% Mn doped ZnO/Au substrate by detecting 140 points at the same place with an interval time of 1 second.

Let us now compare the amplitude of the SERS signals with increasing concentration of the Mn doping. R6G was chosen as the analyte. As shown in Fig. 3a–h, five strong peaks of R6G can be detected in all cases: 1650 cm^−1^, 1511 cm^−1^, 1360 cm^−1^, 1305 cm^−1^, 1186 cm^−1^, the amplitude of which increases monotonically with concentration of Mn, in agreement with the FDTD simulations. The EF was quantified based on the peak centered at 1511 cm^−1^ using the following equation:


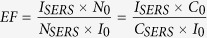


where C_0_ and I_0_ correspond to the concentration and peak intensity, respectively, of the normal Raman measurement with 1 × 10^−3^ M R6G solution on a Si wafer (Figure S6), and the data of C_SERS_ and I_SERS_ were derived from the concentration and peak intensity of a typical SERS measurement with 1 × 10^−8^ M R6G solution. The EF was calculated as (2300 × 1 × 10^−3^)/(80 × 1 × 10^−8^) = 2.8 × 10^6^. Let us point out that a total of 200 points were probed (see Fig. 3i,j), and all exhibited favourable SERS enhancements for the detected molecules, which proved the high uniformity of the substrate. Another key avenue to assess the exceptional SERS uniformity of the substrate is to calculate the relative standard deviation (RSD) of the intensity of the carbon skeleton stretching modes. The two strongest peaks centered at 1360 cm^−1^ and 1511 cm^−1^ were selected as candidates, and the RSD values of these two peaks are 14.4% and 15.6%, respectively, demonstrating suitability for highly sensitive SERS substrate^29^. We also probed the thermal stability of the 8% Mn doped ZnO/Au SERS substrate by detecting 140 points at the same place with an interval time of 1 second (Fig. 3k). Only 6% decrease was produced in this substrate compared to 27% in Si/Au and 36% Si/Cu substrates (Figure S7). It is well-known that heat generated from the laser irradiation on SERS substrates produces unfavorable effects on the sensitivity of SERS detections. The major reason is the limited heat capacity of noble metal Au (25.413 J·mol^−1^·K^−1^), Cu (24.78 J·mol^−1^·K^−1^) and Ag (24.9 J·mol^−1^·K^−1^)^30^. In order to increase thermal stability, one available strategy is to combine noble metal with a higher heat capacity material^31^. ZnO (40.30 J·mol^−1^·K^−1^) owns a much higher heat capacity than those of noble metals that markedly guarantee the thermal stability of Mn doped ZnO/Au SERS substrate.

**Figure 4:**
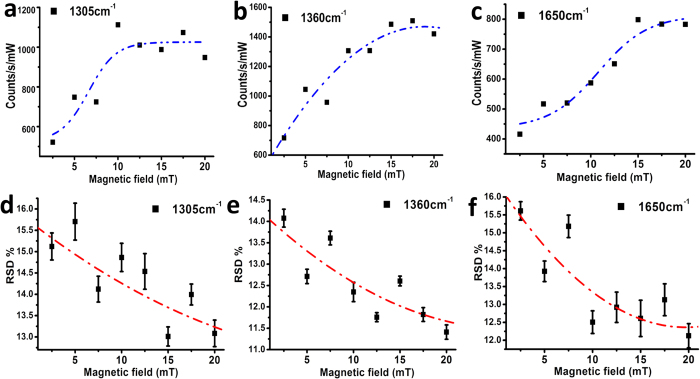
(**a–c**) The changes of intensities of the main Raman vibrations with an increase of external magnetic field (**a**) 1305 cm^−1^, (**b**) 1360 cm^−1^, (**c**) 1650 cm^−1^ and (**d–f**) the changes of RSDs of the main Raman vibrations with an increase of external magnetic field (**d**) 1305 cm^−1^, (**e**) 1360 cm^−1^, (**f**) 1650 cm^−1^.

Considering the room temperature ferromagnetism of nonpolar 8% Mn doped ZnO, it was natural to verify possible effects of an external magnetic field on the SERS signal. The magnetic field was generated by a commercial electromagnet. Surprisingly, the magnetic field presents unprecedentedly observed improvements on the Raman signals. As shown in Fig. 4a–c, the intensities of Raman signals become stronger by increasing the magnetic field. Furthermore, the RSD of all the peaks can be reduced by almost 12% with applied magnetic field as small as 20 mT. No such effect was observed with nanostructured, undoped ZnO/Au substrates having the same morphology. We ascribe this behavior to the formation of magnetic polarons in the DMS under magnetic field, and consequent increase of thermal stability of the SERS substrate.

## Discussion

Diluting magnetic ions, such as Mn, in a semiconductor oxide, such as ZnO, does not trivially lead to a ferromagnetic uniform phase due to direct exchange interaction. Infrared magneto-optical imaging has clearly shown that sub-μm size ferromagnetic domains, namely bound magnetic polarons, are formed around magnetic dopant ions and coexist with the paramagnetic phase, with the density of polarons increasing at the expenses of the paramagnetic phase when an external field is applied^32^. For this reason the M-H loop of a DMS shows little remanence and coercivity, like in Fig. 2e. Although the concept of bound magnetic polaron was first introduced to explain the anomalous magnetism observed in insulating europium oxide (EuO) when doped with the rare-earth ferromagnetic metal gadolinium (Gd), the concept has been extended to diluted magnetic semiconductors^18^. In the latter, magnetic bound polarons are usually formed around oxygen vacancies (V_O_’s), which are double donors and provide the carriers to mediate the interaction between the localized spins of the magnetic dopant ions. The similarities between Gd-EuO and Mn-ZnO can be extended beyond the magnetic behaviour. We have recently demonstrated, in a Report in this same journal, that the complex Mn-V_O_ in ZnO plays the same role as the Gd in EuO^33^. While Gd in EuO replaces Eu^2+^ with valence 3+, therefore providing both the localized *4f* spin and the mediating carrier for the formation of the magnetic polaron, Mn replace Zn^2+^ in ZnO with the same valence, therefore providing only the localized *3d* spin. The formation of the magnetic polaron must rely on mediating carriers provided by the V_O_. In our previous report we have extended the similarities between two these compounds beyond magnetism, showing that they share similar optical and electronic properties. More important, we have demonstrated that they share similar magneto-electric and magneto-optical properties, *i.e.* the physical properties are dependent on the application of an external magnetic field.

In any material, electronic scattering is spin dependent, therefore a magnetic order increases thermal stability. The case of DMSs is somehow peculiar because ferromagnetism in these materials is field-induced. DMSs are double phase systems: a paramagnetic phase, which dominates in zero applied field, coexists with a magnetic phase consisting of bound magnetic polarons, which can be seen as ferromagnetic domains within the paramagnetic phase. In the paramagnetic phase, spin fluctuations significantly contribute to electronic scattering while this contribution becomes negligible in the ferromagnetic domains represented by the magnetic polarons^21^. Since the ferromagnetic phase grows at the expenses of the paramagnetic phase when an external field is applied, the application of a magnetic field has a positive effect on the Raman signal because it reduces the overall thermal fluctuations in the substrate^21^. The magnetic-field induced nucleation of bound magnetic polarons in the substrate results in a signal enhancement and RSD reduction.

Let us finally notice that, for fixed value of magnetic field, the density of magnetic polarons in the film depends on the concentration of magnetic dopant. Therefore doping with a 3*d* magnetic metal has a twofold positive effect on the SERS signal: it increases dielectric constant, which might be as well achieved with non-magnetic metal dopants, and stabilizes the SERS signal, with the stabilization improving under the application of a magnetic field.

## Methods

### SERS Substrates preparation

Single phase dense Zn_1-x_Mn_x_O (x = 0, 0.04 and 0.08) targets were prepared by a standard solid-state reaction method. During the PLD deposition, the wafer temperature was kept at 400 °C. For the pulsed KrF excimer laser (*λ* = 247 nm), the energy was 300 m*J* and the repetition rate was 10 Hz. The films were grown in high vacuum (10^−5 ^mbar) for 20 minutes. Magnetron Sputtering Deposition: Au was deposited on the ZnO in Argon plasma (20 sccm, 80W).

### Characterization

The crystal structure of the films was investigated by X-ray diffractometry using a Philips X’Pert with Cu Kα radiation source. The stoichiometry of both target and films was checked by energy-dispersive X-ray spectroscopy (EDS). Scanning electron microscope (SEM) was utilized to study the surface morphology. XPS measurements were carried out with a 1486.6 eV Al K*α* source. Calibration of the energy scale of the spectrometer was obtained by using the adventitious C 1 *s* peak at 284.9 eV.

### 3D-FDTD Simulation

The penetration of the electromagnetic field of the system was calculated using a 3D-finite difference time domain (FDTD) software. The homogeneous gold monolayer was simulated by the Au NPs with diameter of 8 nm. These particles were periodically arranged in a square array on the Mn doped ZnO thin film with thickness of 250 nm. The distance between Au NPs was 8 nm. A *p*-polarized 633 nm plane wave was placed above the Au array and polarized to the *x*-axis. The mesh size used in the calculations was 0.25 nm × 0.25 nm × 0.25 nm. The simulation time was set at 1000 fs, ensuring the fields to decay completely before termination of the simulation.

### Raman and SERS measurement

Raman spectra was collected by using a HR 800 Raman spectrometer (J Y, France) with a confocal Olympus microscope and a synapse charge-coupled detector. The line mapping mode and 1 *μ*m increment were carried out in the SERS detection. The spectrograph used 600 g·mm^−1^ gratings and an accumulation time of 1 s. LMPlanFl 50 × objective lens was chosen to collect the SERS spectra. A 633 nm He - Ne laser was employed in whole detections.

## Additional Information

**How to cite this article**: Shao, Q. *et al.* Magnetic-Polaron-Induced Enhancement of Surface Raman Scattering. *Sci. Rep.* **6**, 19025; doi: 10.1038/srep19025 (2016).

## Supplementary Material

Supplementary Information
