# Supplementary material for: Magnetic-Polaron-Induced Enhancement of Surface Raman Scattering
Source: Sci Rep. 2016 Jan 12;6:19025. doi: 10.1038/srep19025 (PMC4709511; doi:10.1038/srep19025)
Supplement: Supplementary Information [file srep19025-s1.doc]

Magnetic-Polaron-Induced Enhancement of Surface Raman Scattering

Qi Shao^1,2^, Fan Liao^3^ and Antonio Ruotolo^1,2,*^

^1^ *Department of Physics and Materials Science, City University of Hong Kong, Kowloon, Hong Kong SAR, China*

^2^ *Shenzhen Research Institute, City University of Hong Kong, High-Tech Zone, Nanshan District, Shenzhen 518057, China*

*^3^ Jiangsu Key Laboratory for Carbon-Based Functional Materials and Devices, Institute of Functional Nano and Soft Materials Laboratory (FUNSOM), Soochow University, Suzhou, Jiangsu 215123, China*

**Table S1:** Dielectric constants of different Mn (0%, 2%, 4%, 5%, 6%, 8%) doped ZnO with *λ_l_ =* 633 nm.

| **Mn %** | *ε***1** | *ε***2** |
| --- | --- | --- |
| **0** | 2.5632 | 1.95E-07 |
| **2** | 2.64943 | 3.11E-07 |
| **4** | 2.74167 | 4.42E-07 |
| **5** | 2.79024 | 5.15E-07 |
| **6** | 2.8618 | 4.90E-07 |
| **8** | 3.01649 | 4.29E-07 |


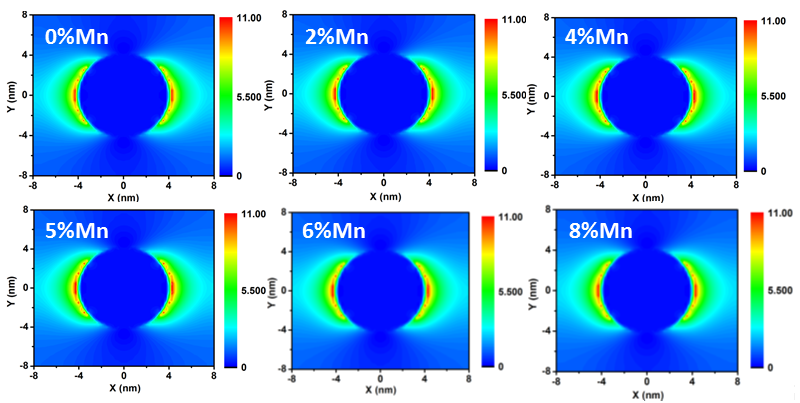


**Figure S1.** Image illustrations of the different intensity (|*E/E_0_*|^2^) distributions between the gold nanoparticles on the different Mn (0%, 2%, 4%, 5%, 6%, 8%) doped ZnO observed on x-y plane.


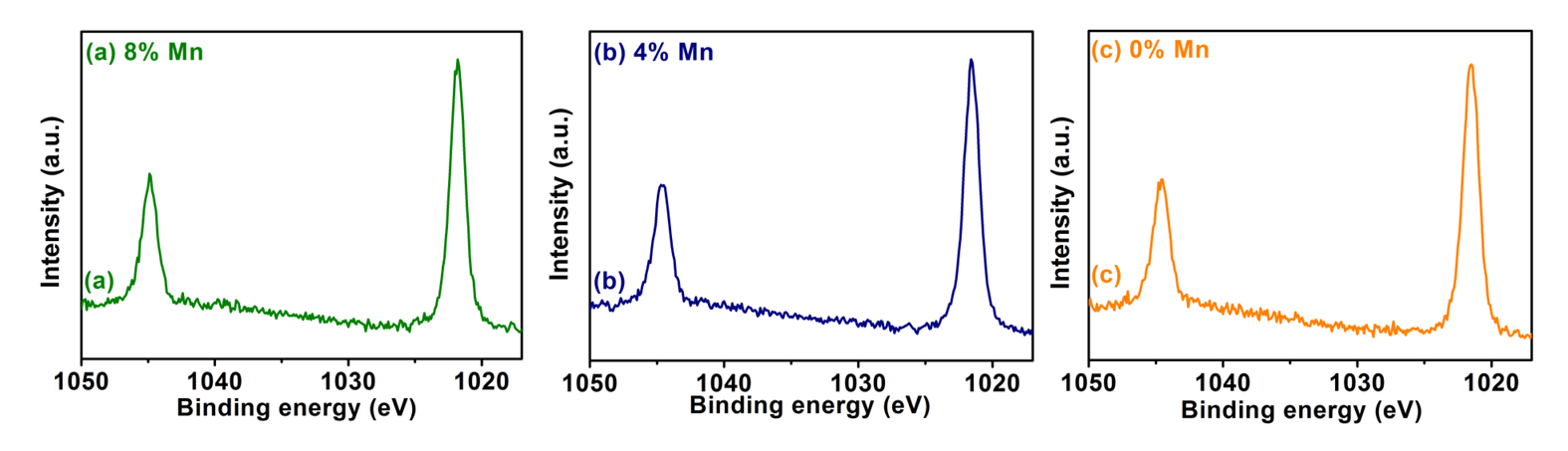


**Figure S2.** XPS spectrums of Zn 2*p* peaks, (a) 8% Mn doped ZnO; (b) 4% Mn doped ZnO; (c) 0% Mn doped ZnO. In order to investigate the surface electronic structures of the film, XPS analyses were carried out. The energy distribution of the emitted core-level electrons observed by Zn was shown in Figure S2a. The peaks located at 1021.42 eV and 1044.60 eV are corresponding to the double spectral lines of Zn 2*p*_3/2_ and 2*p*_1/2_, matched with binding energy of Zn^2+^ in ZnO.^s1^


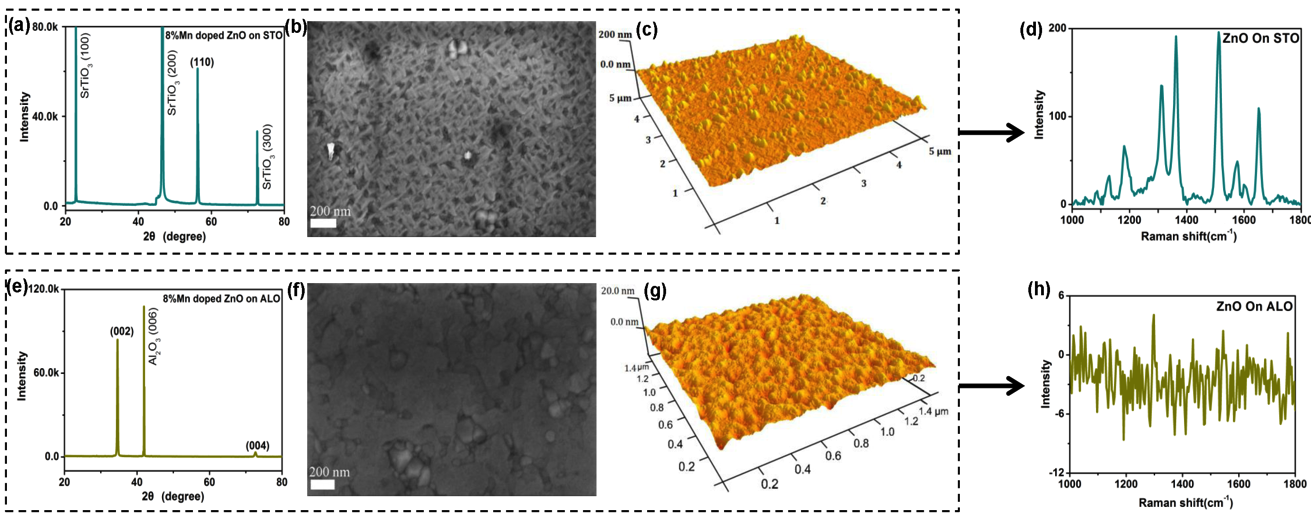


**Figure S3.** Morphology-dependent SERS performance of nonpolar and polar ZnO films. a,e) XRD patterns a) nonpolar ZnO film and e) polar ZnO film. b,f) SEM images of b) nonpolar ZnO film and f) polar ZnO film. c,g) AFM images of c) nonpolar ZnO film and g) polar ZnO film. d,h) SERS detections of 1×10^-8^ R6G solution in d) nonpolar ZnO/Au film and h) polar ZnO/Au film.

A nanostructured substrate is a necessary condition to obtain SERS. In fact, in Fig. S3 we compare the SERS activity of nonpolar, nanostructured ZnO/Au grown on (100) STO with that of polar, smooth ZnO/Au grown on lattice-matched (006) Al_2_O_3_ (ALO). The former shows a roughness of about 32 nm, as measured by atomic force microscope (AFM), whereas the latter shows a roughness of only 3 nm. Owing to its well-established vibrational features, 1 × 10^−8^ M R6G solution was selected as the probe molecule to detect the SERS activity. The SERS detections were probed in a 150 μL quartz cell immersed in 1 × 10^−8^ M R6G aqueous solution. A remarkable SERS intensity was exhibited by the nonpolar SERS substrate fabricated using STO wafer (Fig. S3d, 3h). The main vibrations of R6G solution at 1360, 1511, 1572, and 1650 cm^−1^ were detected, which matched well with previous reports. Some bands centered at 1126 and 1602 cm^−1^, which can hardly be observed in normal Raman spectrum, also depict strong SERS signals, further proving the excellent enhancement of the substrate. However, in the polar SERS substrate with ALO wafer, no evidence of Raman signal was detected because of the smooth surface. A considerable increase in the surface roughness of SERS substrate necessarily enhances the SERS signal under appropriate excitation because it increases the number of electromagnetic hot spots of ZnO/noble metal systems, thereby promoting strong electromagnetic enhancement for the SERS signal.

| *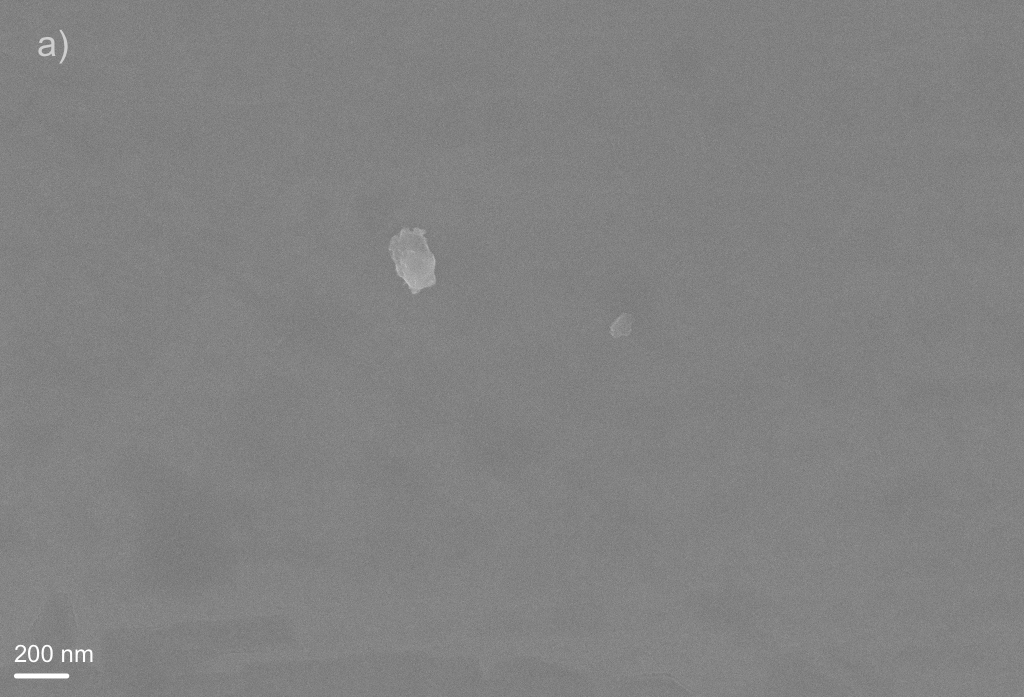* | *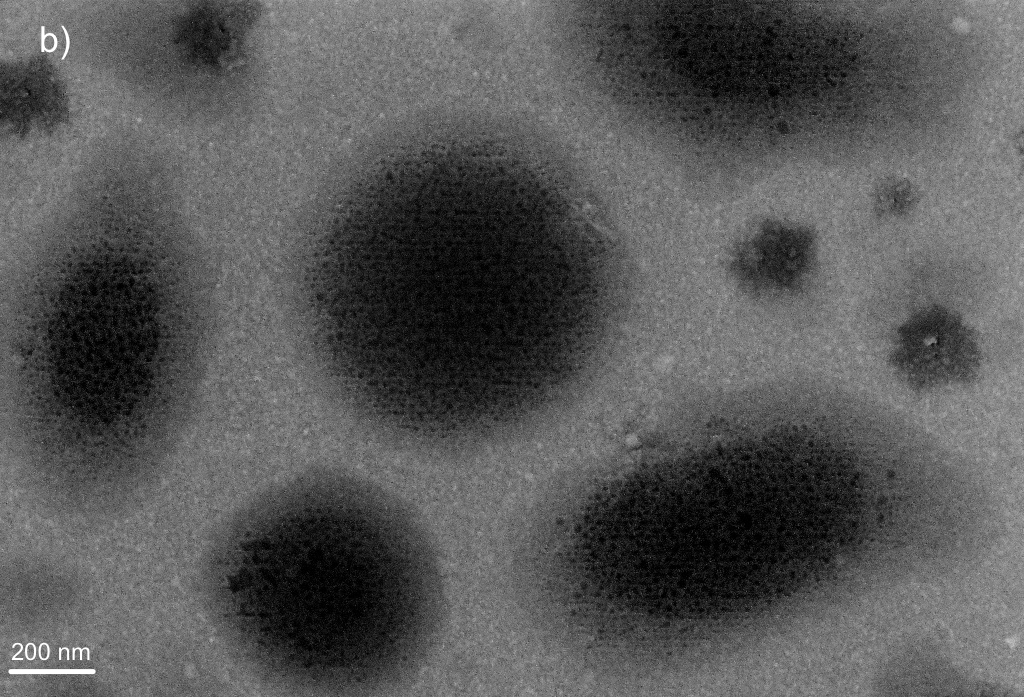* |
| --- | --- |
| **Figure S4.** a) Au sputtered on a continuous, non-nanostructured Mn-ZnO film and b)  Au sputtered under the same conditions on a nanostructured Mn-ZnO film. In b) Au clusters in nanoparticles in the valleys. | |

**
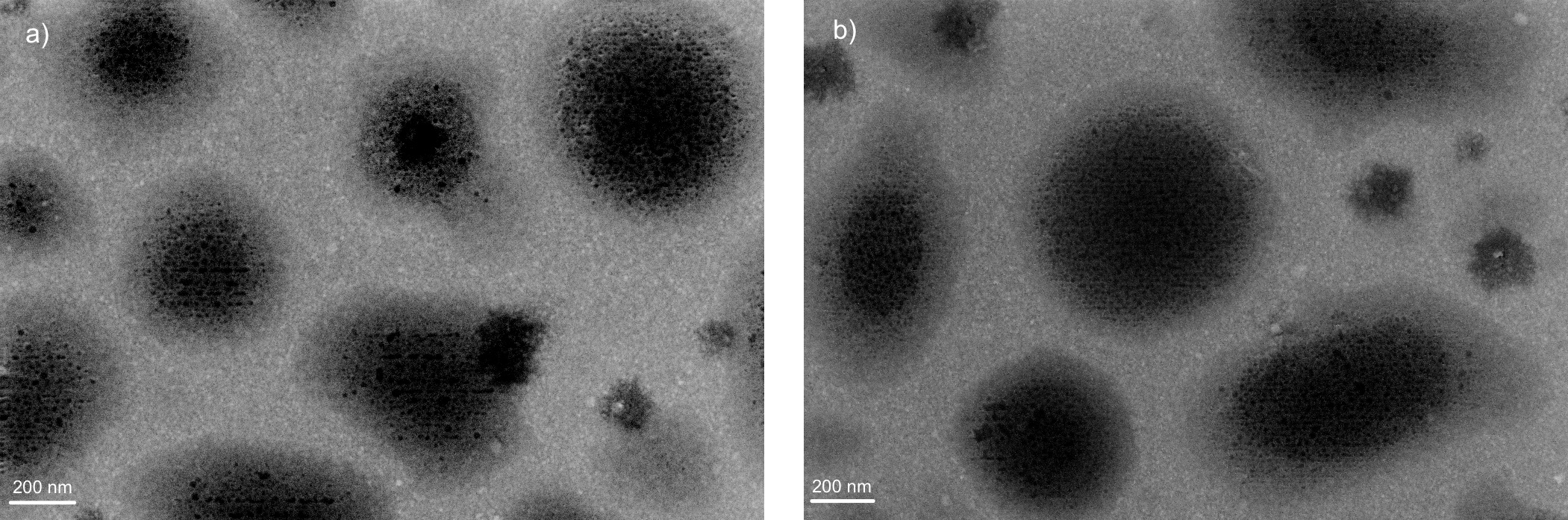
**

**Figure S5.** SEM images of (a) 4% and (b) 8% Mn doped ZnO after sputtering of Au.


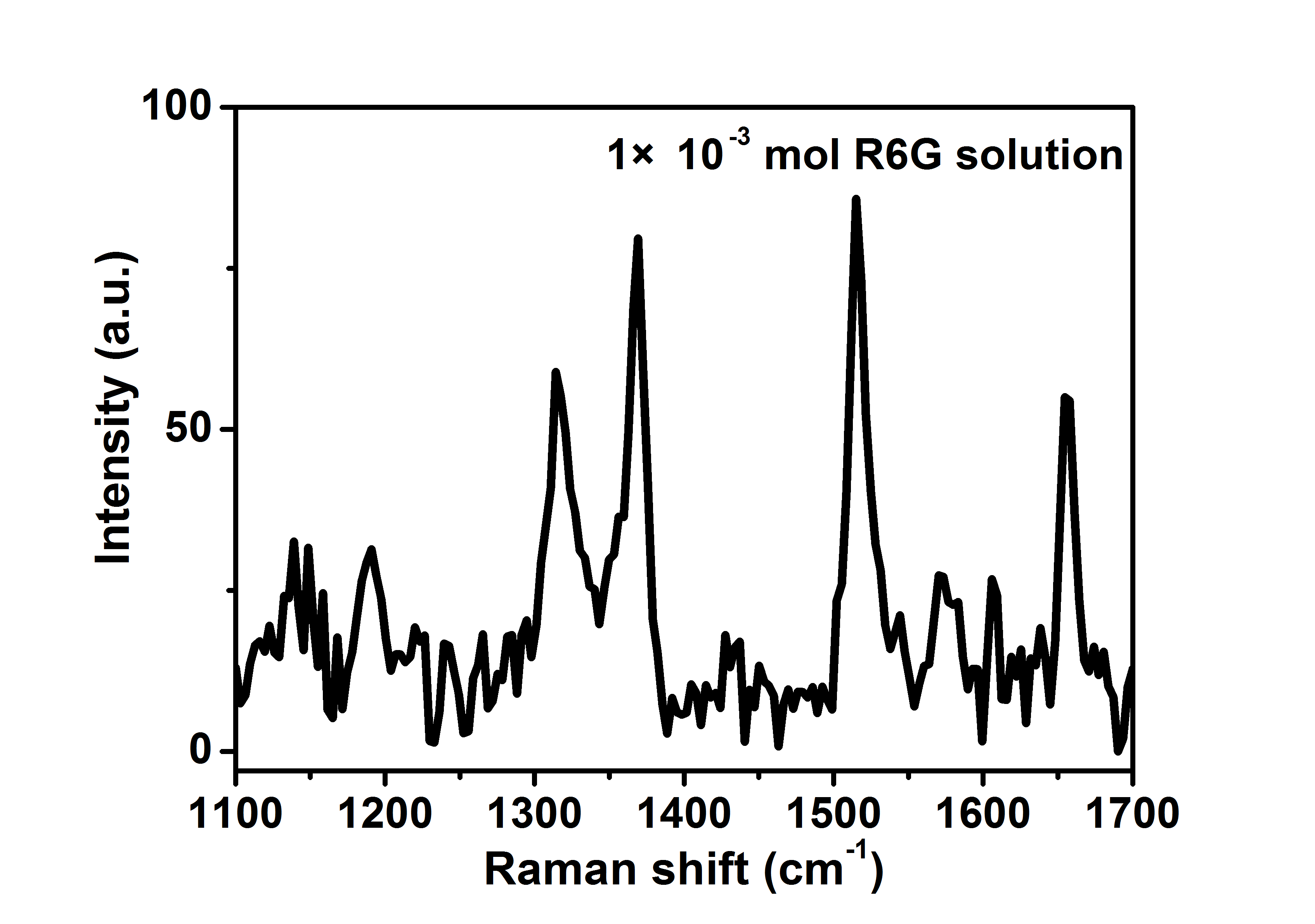


**Figure S6.** The normal Raman spectrum of 1×10^-3^ R6G solution.

**(a)**
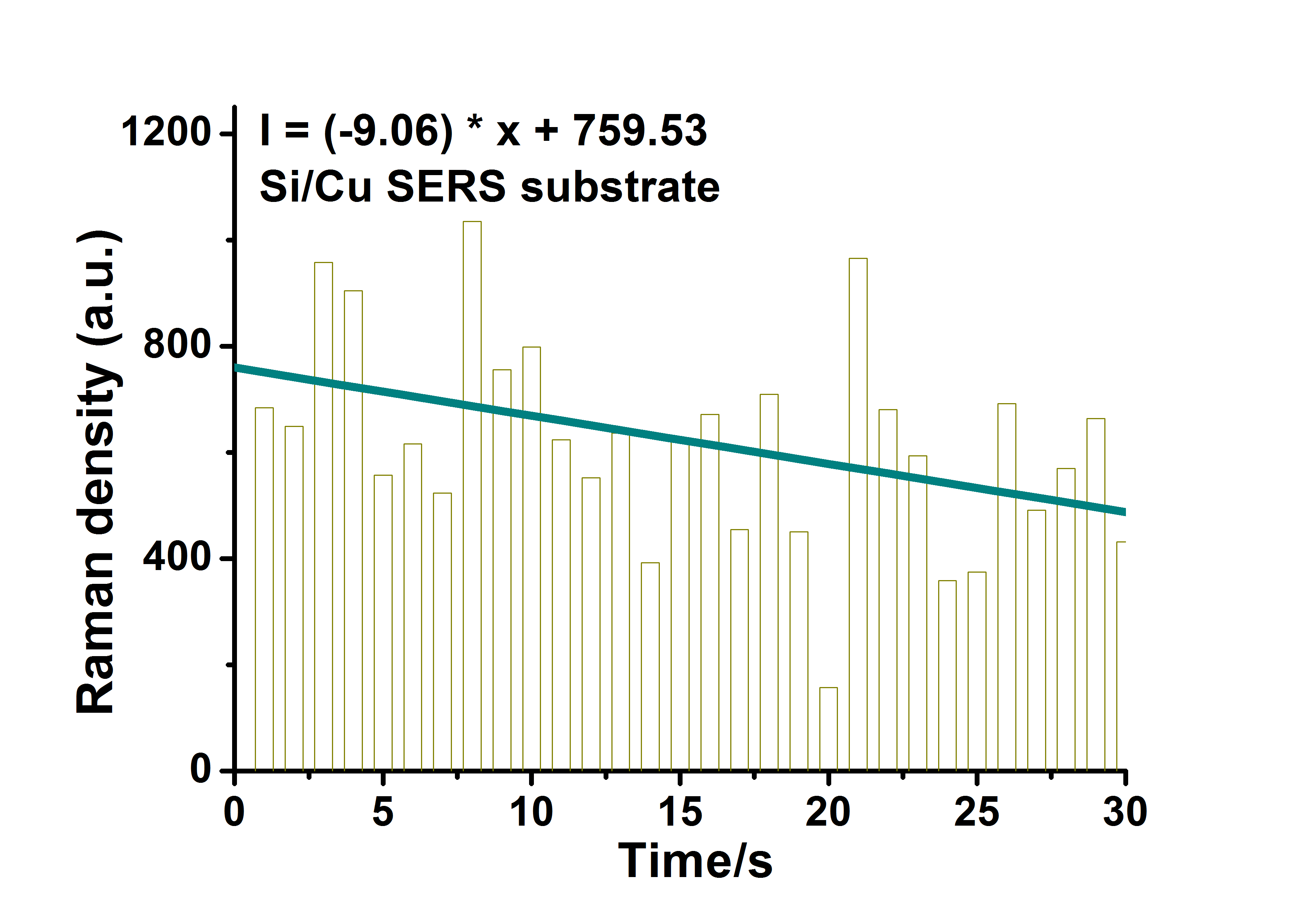


**(b)**
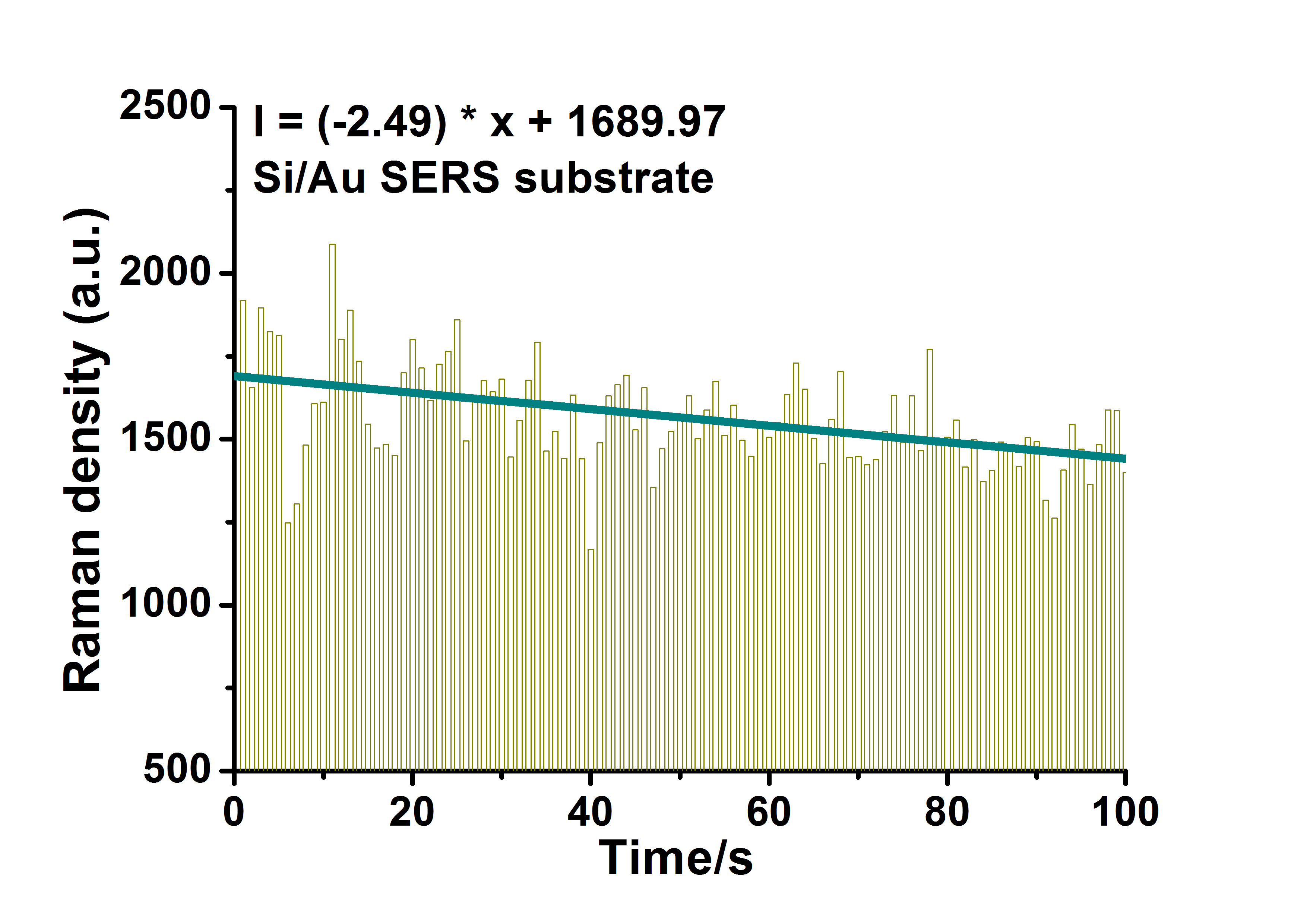


**Figure S7.** Intensities of 1360 cm^-1^ to measure thermal ability of (a) Si/Au and (b) Si/Cu substrates by detecting at the same place with an interval time of 1 second.


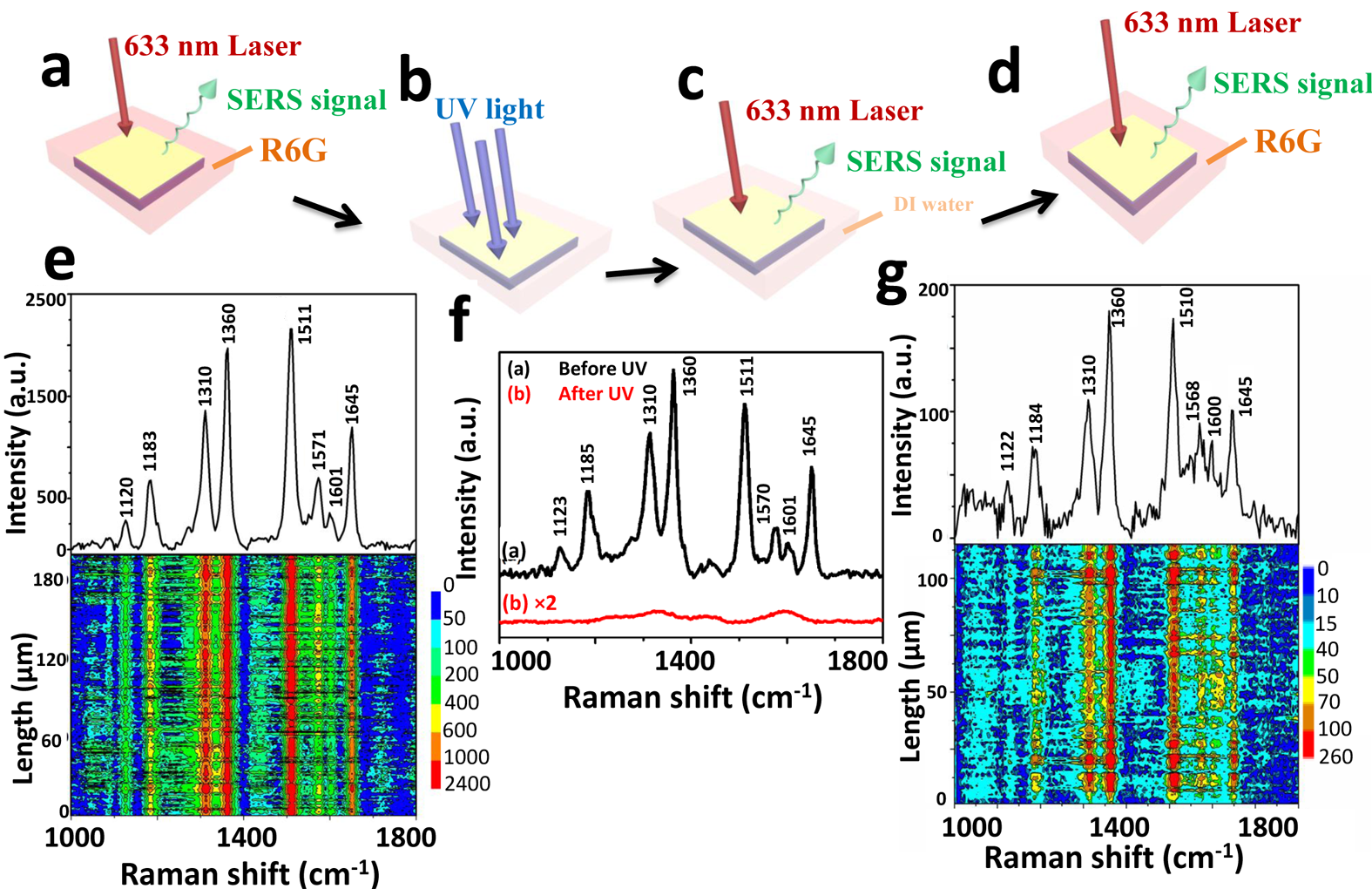


**Figure S8.** (a-d) Scheme of reversible SERS behavior of nonpolar ZnO/Au SERS substrate. (e) Raman spectrum of 1 × 10^−8^ R6G solution on nonpolar 8% Mn-doped ZnO/Au substrate (upper part) and SERS contour (lower part). (f) Comparison of different spectra: SERS spectrum of 1 × 10^−8^ R6G solution before and after UV irradiation. (g) New Raman detection of 1 × 10^−7^ R6G solution after self-cleaning by UV-vis light irradiation on nonpolar 8% Mn-doped ZnO/Au substrate (upper part) and SERS contour (lower part).


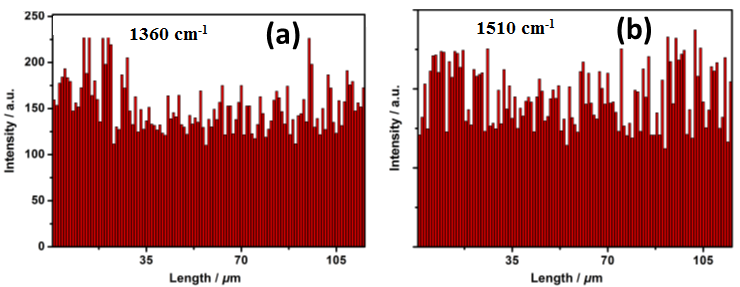


**Figure S9.** The intensities of the main Raman vibrations of 1×10^-7^ M R6G solution after UV irradiation, a) 1360 cm^-1^, b) 1510 cm^-1^.

References

[S1] J. F. Moulder, W. F. Stickle, P. E. Sobol, and K. D. Bomben, in Handbook of X-Ray Photoelectron Spectroscopy 89, edited by J. Chastain and R. C.Kings, Jr. Physical Electronics, Eden Prairie, MN, **1995**.
